# Introducing a new concept: Psychological capital of older people and its positive effect on mental health

**DOI:** 10.3389/fpsyg.2023.1083077

**Published:** 2023-03-20

**Authors:** Yaping Xin, Dan Li

**Affiliations:** School of Public Administration, Sichuan University, Chengdu, Sichuan, China

**Keywords:** older people, psychological capital, mental health, reliability, validity

## Abstract

**Objective:**

This study aimed to explore the structure of psychological capital (PsyCap) and its positive effects on mental health among older people.

**Methods:**

Study 1 used grounded theory to analyze the semi-structured interviewing data of 17 Chinese older people (60–96 years old) to develop a primary PsyCap questionnaire for older people. Study 2, respectively, applied exploratory factor analysis (EFA) with 198 Chinese older people (*M* = 69.2; *SD* = 6.685) and confirmatory factor analysis (CFA) with 370 Chinese older people (*M* = 73.84; *SD* = 9.416) to test a seven-factor structure for PsyCap. Study 3 used 328 participants (*M* = 79.73; *SD* = 9.073) to examine the correlation between PsyCap and mental health.

**Results:**

Study 1 identified that PsyCap of older people contains ‘resilience,’ ‘self-efficacy,’ ‘optimism,’ ‘ease and content,’ ‘gratitude and dedication, ‘wisdom,’ and ‘meaning in life’ and generated a primary seven-factor questionnaire. Study 2 proved the overall and internal structure reliability of PsyCap were good (Cronbach’s alphas ranged 0.809 ~ 0.935), and the seven-factor measurement model fitted the data well (*χ*^2^/*df* = 2.07, RMSEA = 0.05, RMR = 0.05, CFI = 0.95, IFI = 0.95, TLI = 0.94, NFI = 0.91). The PsyCap scale was also proved to an excellent convergent validity, discriminant validity, calibration validity, and measurement invariance across different groups. Study 3 found that PsyCap and its seven factors significantly correlated with depression (*r* = −0.419 ~ −0.163, *p* < 0.01) after controlling the demographic variables.

**Conclusion:**

These findings provide a reliable and valid assessment for quantitative empirical research of PsyCap among older people and show significant impacts on mental health among older people, which offers new insight into improving mental health from the perspective of positive psychology.

## Introduction

Psychological capital (PsyCap) refers to ‘an individual’s positive psychological state of development’ that includes various positive psychological elements, which brings individuals strong competitive advantages by investing and developing ‘who you are’ (Luthans et al., 2007). As a higher-order construct of psychological resources, PsyCap plays an vital role in improving mental health, well-being, and life satisfaction (Luthans et al., 2013; Azimi, 2014; Youssef-Morgan and Luthans, 2015) and also decreasing personal stress, anxiety, and depression (Avey et al., 2011; Rahimnia et al., 2013). It can be seen that PsyCap has positive effects on improving individual mental health, which provides a new theoretical perspective (i.e., positive psychology) to improve older adults’ mental health and social development.

World Health Organization (2015) has emphasized the importance of older adults’ intrinsic capacity (including positive psychological factors) on physical and mental health in the *World report on ageing and health*. However, a latest scoping review have analyzed that existing studies focus more on effects of physiological and social factors on mental health of older people (Murniati et al., 2022). Only some studies have found that positive affect, personality traits, and psychological strength are associated with mental health of older people (Steptoe et al., 2008; Windsor et al., 2015; Bedaso and Han, 2021), while more other positive psychological resources get little attention. As the psychosocial developmental theory suggested, the psychological stage of old age is characterized by using all the positive psychological elements (wisdom, optimism, resilience, autonomy, self-esteem, etc.) to balance negative elements from the past and future (shame, fear, despair, regret, etc.) to achieve ego integrity (Erikson et al., 1994). Therefore, this study aims to introduce a new concept (i.e., PsyCap) to reflect the inner positivity of older people and enrich the literature on improving their mental health.

PsyCap has been widely concerned with organizational behaviors and management as an essential resource beyond human and social capital. In early research, the measurement of PsyCap mainly revolves around various personality traits, like self-esteem, self-efficacy, control points, emotional stability, neuroticism, extroversion, openness, affinity, and responsibility (Goldsmith et al., 1997; Letcher, 2003; Cole, 2006). With the rise of positive organizational behavior (POB), studies gradually focus on individuals’ positive psychological states, such as hope, optimism, and resilience (Luthans et al., 2005). Furthermore, POB still developed a set of strict scientific standards for screening PsyCap components that must meet the scientific criteria of being theory and research-based, measurable, state-like or developmental, and related to work performance outcomes (Avey et al., 2010). Following the standards of POB, Luthans et al. (2007) and his group developed a 24-item PsyCap Questionnaire (PCQ-24) to measure hope, optimism, resilience, and self-efficacy (HERO) of employees. Many studies have tested the reliability and effectiveness of PCQ-24 and also found its positive effects on promoting work satisfaction, organizational behaviors, and work performance (Nolzen, 2018; Donaldson et al., 2020).

As Table 1 presented, some scholars have found different PsyCap components in other research objects in workplace, and these new PsyCap scales have good reliability and validity. For the organizational managers, mindfulness also had a same positive effects to relieve their anxiety, depression, and cynicism as HERO (Roche et al., 2014). Studies of entrepreneurs suggested that their PsyCap also included courage, wisdom, innovation, and flexibility, which positively impact various dimensions of entrepreneurial success (Bockorny and Youssef-Morgan, 2019). Similarly, an expanded PsyCap archetype of sports employees, labeled A-HERO (A means authenticity), was explored and examined in American sports organizations (Kim et al., 2021). Besides, studies also found four other elements of PsyCap (i.e., tolerance, respect, modesty, and dedication) among Chinese college teachers (Bin and Zhu, 2020).

**Table 1:** PsyCap scales of different research objects in the previous studies.

Authors	Objects	PsyCap components	Reliability	Correlated variables
Luthans et al. (2007)	Employees	Hope, optimism, self-efficacy, and resiliency	Cronbach’s α was 0.881	Work attitudes, behaviors, and performance
Roche et al. (2014)	Managers	Mindfulness, hope, efficacy, resilience, and optimism	Cronbach’s α was 0.840	Anxiety, depression, and cynicism
Bockorny and Youssef-Morgan (2019)	Entrepreneurs	Courage, hope, efficacy, resilience, and optimism	Cronbach’s α was 0.812	Entrepreneurial success
Ren and Ji (2019)	Adolescents	Resilience, optimism, tolerance, gratitude, self-efficacy	Cronbach’s α was 0.880	Perceived social support and loneliness
Xu and Yu (2019)	Disabled people	Self-acceptance, hope, optimism, and resilience	Cronbach’s α was 0.860	Employability
Bin and Zhu (2020)	College teachers	Tolerance, respect, modesty, dedication, confidence, hope, and resiliency	Cronbach’s α was 0.955	/
Kim et al. (2021)	Sport employees	Authenticity, hope, efficacy, resilience, and optimism	Cronbach’s α was 0.862	Creative work behaviors
Xu et al. (2021)	Volunteers	Responsibility, gratitude, self-efficacy, resilience, and hope	Cronbach’s α was 0.857	Volunteering motivation and behaviors

On the other hand, some studies shifted the focus from the workplace and began to pay attention to PsyCap and the social development of other people. For example, tolerance and gratitude played a same role in improving perceived social support and decreasing loneliness of adolescents as resilience, optimism, and self-efficacy (Ren and Ji, 2019). The PsyCap of volunteers was composed of responsibility, gratitude, self-efficacy, resilience, and hope, and these resources significantly affected their voluntary motivation and behaviors (Xu et al., 2021). In addition, studies found self-acceptance was a special element of PsyCap among disabled people, which helps them improve employability (Xu and Yu, 2019). These studies have demonstrated that the PsyCap theory has been applied to non-organization fields, and its dimensions and measurements vary across different groups and cultures.

Although existing studies have developed various types of PsyCap scales for different groups of people, there is no available scale for measuring PsyCap in older age groups. There are two reasons for developing a new PsyCap scale for Chinese older people rather than adapting existing scales. First, older adults are isolated from the formal social organization by the retirement system, which leads to great changes in individual psychological characteristics and behaviors (Riley and Riley, 1994; Hagestad and Uhlenberg, 2005). Some special positive resources of older people, including the sense of mastery and coherence, expectancy of control, and spiritual intelligence (Wells and Kendig, 1999; Windle et al., 2008; Sargent-Cox et al., 2012; Jafari and Hesampour, 2017) cannot be measured by those PsyCap scales based on work (study) performance. Second, this study focuses on Chinese older people, and it is necessary to consider the variations of PsyCap elements and scales across different cultural backgrounds. Studies have revealed that wisdom and gratitude are two unique dimensions of the PsyCap construct in Asian culture (Ashraf and Khan, 2017), while spirituality and courage are considered PsyCap elements in African culture (Dhir and Sharma, 2020). Therefore, this study plans to develop a new effective measurement tool to measure PsyCap of Chinese older people and examine the positive effect of PsyCap on mental health.

## Method and results

This study included three parts. Study 1 aimed to use the method of grounded theory to identify the components of PsyCap for Chinese older people and generate a pool of items that measure the dimensions of older people’s PsyCap. Study 2 planned to examine the initial factor structure of PsyCap for Chinese older people and test the reliability and validity of the PsyCap questionnaire through quantitative research. Study 3 was used to examine the relationship between PsyCap and mental health.

## Study 1

### Participants

This study adopted theoretical sampling and semi-structured interviews to collect in-depth qualitative data about the psychological strengths of Chinese older people and finally selected 17 participants with positive performances in psychological status, behaviors, cognition, and social life. The average age of participants was 75.5 years old, and their ages ranged from 60 to 96 years old. 41.2% of them were male, and 58.8% were female. According to their education level, 47.1% of participants were in senior high school and above. Their pre-retirement occupations included professors, civil servants, researchers, soldiers, engineers, teachers, nurses, workers, and farmers.

### Measurements

The semi-structured interviews usually used open-ended questionnaires. This study focused on the following questions about older people: (a) How did you adjust to the life changes around retirement, including health, interpersonal relationships, income, time use, and life goals? (b) How did you think about and deal with the troubles, challenges, or difficulties in your retired life? (c) What are psychological strengths important to help you maintain a positive life? (d) What do you think about being older, getting diseases, losing functional abilities, and even facing death? The interviews lasted 45 to 90 min.

### Procedures

This study first finished coding analysis of the qualitative data based on the grounded theory, which was used to compare incidents and elements, integrate categories, and construct theory from the investigation data (Shah and Corley, 2006). Corbin and Strauss (1990) divided the coding process into three steps: open coding, axial coding, and selective coding. Following these three kinds of coding analysis, this study used the software of Nvivo 11 to identify critical components of PsyCap among Chinese older people. Based on these results, this study invited some experts in the field of psychology and gerontology to generate a pool of items that measure the dimensions of older people’s PsyCap and compiled a primary PsyCap questionnaire.

### Results

PsyCap of Chinese older people refers to the measurable and exploitable psychological resources promoting older adults’ health and social development. Results of coding analysis found that the PsyCap structure contained seven elements: (1) Resilience: adapting to new changes and bouncing back from adversity (Luthans, 2002). (2) Self-efficacy: keeping confidence to finish some challenging tasks in daily life (Stajkovic and Luthans, 1998). (3) Optimism: making positive expectations for the future (Luthans et al., 2007). (4) Contentment and happiness: perceiving the present situation as enough and entire and then generating true happiness in spirit (Cordaro et al., 2016). (5) Gratitude and dedication: feeling grateful for others’ help (gifts) and helping others selflessly (Emmons et al., 2019). (6) Wisdom: handling things with rich knowledge and performing rationally (Luthans et al., 2007). (7) Meaning in life: perceiving self-importance and understanding life goals and missions (Steger et al., 2006).

Based on the results of qualitative analysis, this study generated a pool of items that measure the dimensions of older people’s PsyCap by referring to some mature scales of positive psychology (Webster, 2003; Steger et al., 2006; Ke et al., 2009; Zhang et al., 2010). An expert group in psychology and gerontology were invited to review and revise the appropriateness of the dimension construction and item selection of the PsyCap questionnaire for Chinese older people. Finally, the initial version of the PsyCap questionnaire includes seven dimensions, each with six items of questions. Each question is a self-report item and is scored on a Likert five-point scale.

## Study 2

### Participants

This study carried out two rounds of random sampling surveys with the permission of the Civil Affairs Bureau of provincial capital cities (including Chengdu, Xi ‘an, and Kunming) in western China. All the respondents were over 60 years old and could answer all the questions. Participants provided their informed consent and volunteered to participate in this interview without compensation. The basic information of participants in the two waves was showed in Table 2.

**Table 2:** Demographic characteristics of participants.

Variables		*N* (%)/Mean ± SD		
		Sample 1 (*n* = 198)	Sample 2 (*n* = 370)	Sample 3 (*n* = 328)
Age	Mean ± SD	69.2 (6.685)	73.84 (9.416)	79.73 (9.073)
Gender	Male	40.9%	44.6%	47%
	Female	59.1%	55.4%	53%
Marriage	Single	23.2%	37.8%	41.2%
	Married	76.8%	62.2%	58.8%
Education	Undergraduate and above	27.3%	21.4%	14.4%
	Junior college	16.2%	12.7%	7.6%
	Senior high school	14.1%	14.6%	14.9%
	Junior high school	24.2%	20.3%	13.4%
	Primary school and illiterate.	18.2%	31.1%	49.7%

Sample 1 was used to finish the exploratory factor analysis (EFA) which covered 200 older people and screened out 198 valid questionnaires. The average age of participants was 69.2 years old, with their age range of 60-92 years. 40.9% of participants were male, and 59.1% were male. 76.8% of participants had a couple, and 23.2% were single in marriage. As for educational level, 27.3% of participants were in undergraduate and above, 16.2% were in junior college, 14.1% were in senior high school, 24.2% were in junior high school, and 18.2% were in primary school and bottom.

Sample 2 was used to finish the confirmatory factor analysis (CFA) and covered 400 older people, and screened out 370 valid questionnaires. The average age of participants was 73.84 years old, and their age range was 60–99 years. 44.6% of participants were male, and 55.4% were female. 62.2%of participants had a couple, and 37.8% were single in marriage. As for educational level, 21.4% of participants were in undergraduate and above, 12.7% were in junior college, 14.6% were in senior high school, 20.3% were in junior high school, and 31.1% were in primary school and illiterate.

### Measurements

*Psychological capital* was measured by the initial PsyCap questionnaire constructed in Study 1, including seven dimensions. Each dimension had six self-reported questions, and every question was evaluated by a Likert 5-point scale, from ‘very disagreeable’ to ‘very agreeable.’ The Cronbach’s alpha for *psychological capital* was 0.927.

*Depression* was measured using the shortened version of the Center for Epidemiologic Studies Depression Scale, which comprises nine questions (CES-D9, Santor and Coyne, 1997). Participants were required to evaluate their feelings and emotions in the past week, such as ‘did you feel sad?’, ‘was your sleep restless?’ etc. All these questions had three choices given to the respondents: ‘hardly ever,’ ‘sometimes,’ and ‘often,’ measured by scores 1, 2, and 3, respectively. This study used a total score of nine questions to measure *depression*. The Cronbach’s alpha for *depression* was 0.795.

Single-item self-rated health is a cost-effective measurement technique that reflects older people’s disease/functional health status (Meng et al., 2014). Thus, this study used the question, ‘How do you rate your health at present?’ to measure older people’s *self-rated health*. There were five levels of subjective health status: ‘very unhealthy,’ ‘unhealthy,’ ‘moderately healthy,’ ‘healthy,’ and ‘very healthy,’ measured by scores 1, 2, 3, 4, and 5, respectively.

Studies have demonstrated that the single-item life satisfaction measures performed very similarly to the multiple-item satisfaction with life scale (Cheung and Lucas, 2014). Thus, this study measured older people’s *life satisfaction* by the question, ‘How do you rate your life at present?’ There were five levels of subjective life satisfaction: ‘very unsatisfied,’ ‘unsatisfied,’ ‘moderately satisfied,’ ‘satisfied,’ and ‘very satisfied,’ measured by scores 1, 2, 3, 4, and 5, respectively.

*Social participation* refers to a person’s involvement in activities that interact with others, which can be assessed by questions focusing on the frequency of participation in social activities (Howrey and Hand, 2019). This study used the question, ‘How often do you participate in social activities (including family life, social contact, hobby and sport, volunteering and civic activities, etc.)?’ This question had five choices given to the respondents: ‘every day, ‘not every day, but once a week at least,’ ‘not once a week, but once a month at least,’ ‘not once a month, but once 3 months at least,’ and ‘never,’ measured by scores 1, 2, 3, 4, and 5, respectively.

### Analytical approaches

SPSS 21.0 software and AMOS 21.0 software were used for statistical analysis. Among them, SPSS 21.0 software was used to finish the exploratory factor analysis, reliability analysis, and criterion-related validity test, and AMOS 21.0 software was used to complete the confirmatory factor analysis.

*EFA* is an excellent tool to reveal the interrelations among more extensive measures of observed attributes and extract a smaller number of common factors to represent these interrelations (Fabrigar and Wegener, 2011). These interrelations essentially represent some internal attributes that are unobservable characteristics of people on which people differ in extent or degree, which also can be called latent variables (Tucker and MacCallum, 1997). Therefore, this study adopted the principal component analysis model and Sample 1 to complete the EFA to assess the construct validity to identify the critical components of PsyCap. Measurement items generally can be revised according to the results of EFA.

*CFA* is a type of structural equation modeling (SEM) that deals specifically with measurement models, that is, the relationships between observed measures or indicators (e.g., test items, test scores, behavioral observation ratings) and latent variables or factors (Brown and Moore, 2012). Therefore, this study adopted the SEM and Sample 2 to complete the CFA to assess the construct validity to examine the structure of PsyCap.

### Results

#### EFA

The reliability analysis of Sample 1 showed that the scale’s overall Cronbach’s Alpha was 0.927 (α > 0.7), which meets the reliability test standard. The results of the Bartlett spherical test were significant (*χ*^2^ = 3264.819, *p* < 0.000, DF = 351), and the value of KMO (KMO = 0.870) is larger than 0.7. These results show that the scale is suitable for the EFA of 42 items. Thus, this study used principal component analysis and the maximum variance method to finish EFA. To get a more balanced and better factor structure, this study excluded 14 items whose factor load was less than 0.6 and whose cross-factor load was larger than 0.4. The remaining 28 items were put into the model of EFA again, and the final results generated seven factors: meaning in life, contentment and happiness, optimism, resilience, wisdom, gratitude and dedication, and self-efficacy. The results of the Bartlett spherical test were significant (*χ*^2^ = 3392.305, *p* < 0.000, DF = 378), and the value of KMO (KMO = 0.874) is larger than 0.7. The total explanatory variation of 7 factors was 72.971%, and other indexes (e.g., factor loading, commonality, and rotated eigenvalues) were shown in Table 3.

**Table 3:** Results of the principal components analysis of 28 items (N = 198).

Items	Factors						
	1	2	3	4	5	6	7
I’m always looking to find my life’ purpose	0.894						
I’m searching for meaning of my life	0.864						
I’ve found something that makes my life feel meaningful	0.844						
I’ve discovered a satisfying goal of my life	0.781						
At this point in my life, I find it easy to laugh at my mistakes		0.824					
I prefer just to let things happen rather than try to understand why they turned out that way		0.813					
I can chuckle at personal embarrassments		0.799					
It is better not to know too much about things that cannot be changed		0.791					
I can always see the bright side of things			0.831				
I expect good ending for everything			0.822				
I think life is always good			0.711				
I think there are many good people in society			0.693				
When I feel stressed, I cannot sleep well or eat well				0.848			
When things are going bad, I tend to get down in the dumps				0.844			
Bad experiences easily leave me depressed for a long time				0.759			
I feel myself very tired of living				0.737			
Reviewing my past helps gain perspective on current concerns					0.833		
Reliving past accomplishments in memory increases my confidence for today					0.815		
Recalling my earlier days helps me gain insight into important life matters					0.814		
I reminisce quite frequently					0.625		
I’m so grateful for what life has given me						0.761	
I’ll find a way to give back to those who helped me						0.715	
I believe the value of life lies in dedication						0.770	
Solving the problems of my family is my duty						0.710	
I can always take care of myself							0.790
I think I have enough power to cope with problems							0.770
Most of the time, I feel confident							0.710
I always keep my life organized							0.615
Eigenvalues	9.067	3.008	2.464	2.197	1.465	1.158	1.075
Explain the percentage of variation	32.381	10.742	8.802	7.837	5.232	4.137	3.839
Factor name	Meaning in life	Contentment and happiness	Optimism	Resilience	Wisdom	Gratitude and dedication	Self-efficacy

#### CFA

**Figure 1:**
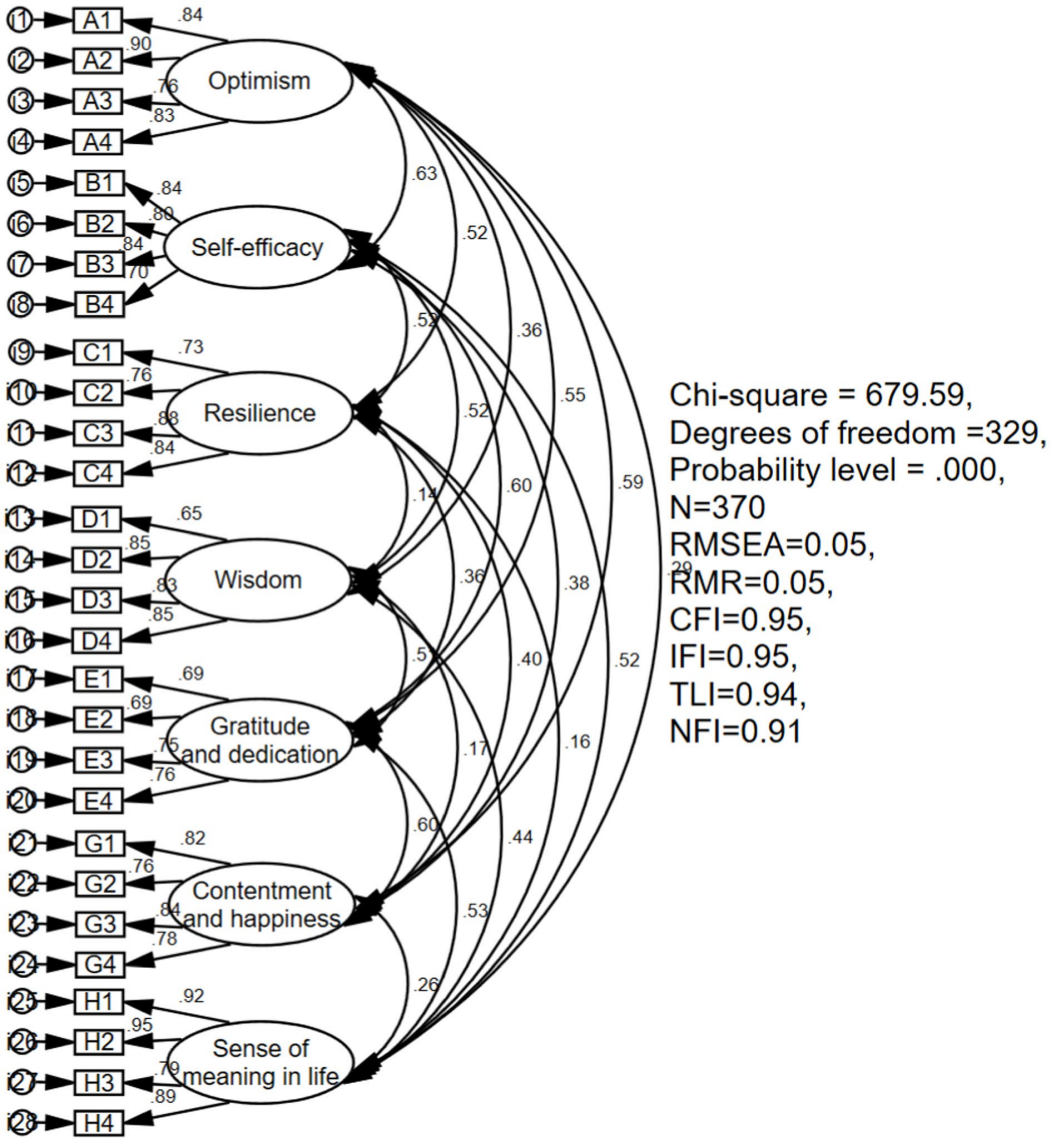
Results of SEM for psychological capital of older people. RMSEA, root-mean-square error of approximation; RMR, root mean square residual; CFI, comparative fit index; IFI, incremental fit index; TLI, Tucker-Lewis Index; NFI, normed fit index.

Based on the EFA, this study set SEM to finish the CFA of the revised questionnaire using Simple 2. The results of SEM (in Figure 1) with seven factors showed that *χ*^2^/*df* was between 2 and 3 (*χ*^2^/*df* = 2.07), the value of root-mean-square error of approximation (RMSEA) was more than 0.08 (RMSEA = 0.05), the value of root mean square residual (RMR) was less than or equal to 0.05 (RMR = 0.05), the values of comparative fit index (CFI), incremental fit index (IFI), Tucker-Lewis index (TLI), and normed fit index (NFI) were more than 0.9 (CFI = 0.95, IFI = 0.95, TLI = 0.94, NFI = 0.91). Thus, this measurement model provided a good fit for the observed data. In addition, each item had a high load on the corresponding factors (the standardized load is between 0.65 ≤ 0.95, and the value of *p* < 0.001).

#### Reliability

Internal consistency for the total scale usually can be evaluated by Cronbach’s alpha (Polit and Beck, 2010). Thus, this study adopted the reliability analysis model to finish the reliability analysis of questionnaires. Based on the EFA, the revised questionnaire covered seven factors and 28 items generated. Reliability analysis results showed that the overall Cronbach‘s alpha of the revised PsyCap scale was 0.931 (α > 0.7). Meanwhile, Cronbach‘s alphas of meaning in life, contentment and happiness, optimism, resilience, wisdom, gratitude and dedication, and self-efficacy were 0.935, 0.875, 0.897, 0.876, 0.869, 0.809, and 0.870, respectively (shown in Table 4). All the reliability coefficients were higher than 0.7, which indicates that the overall reliability and internal structure reliability of PsyCap among older people are good.

**Table 4:** Results of reliability and validity of the revised PsyCap scale (*N* = 370).

	Items	Standardized loadings	AVE	CR	Cronbach’s alpha	The overall Cronbach’s alpha
Optimism	O1	0.840**	0.693	0.900	0.897	0.931
	O2	0.895**				
	O3	0.760**				
	O4	0.828**				
Resilience	R1	0.725**	0.646	0.879	0.876	
	R2	0.764**				
	R3	0.882**				
	R4	0.835**				
Wisdom	W1	0.650**	0.639	0.875	0.869	
	W2	0.847**				
	W3	0.829**				
	W4	0.853**				
Gratitude and dedication	G1	0.692**	0.523	0.814	0.809	
	G2	0.688**				
	G3	0.747**				
	G4	0.763**				
Contentment and happiness	C1	0.815**	0.638	0.875	0.875	
	C2	0.758**				
	C3	0.842**				
	C4	0.776**				
Meaning in life	M1	0.918**	0.788	0.937	0.935	
	M2	0.948**				
	M3	0.789**				
	M4	0.887**				
Self-efficacy	S1	0.836**	0.633	0.873	0.87	
	S2	0.801**				
	S3	0.839**				
	S4	0.698**				

*Represents a significance level of *p* < 0.05; **Represents a significance level of *p* < 0.01. AVE, the average variance extracted; CR, construct reliability.

Measurement invariance refers to members of different populations with the same standing on the measured construct receiving the same observed score on the test, which is generally assessed by the method of CFA (Schmitt and Kuljanin, 2008). Therefore, this study set a multi-group SEM to test the measurement invariance of the revised PsyCap scale across male (*N* = 165) and female (*N* = 205) groups. As Table 5 presented, all the models fit the observed data well. Results showed that model 1 (*p* = 0.519) and model 2 (*p* = 0.428) rejected the group difference hypothesis, and model 3 (*p* = 0.009) and model 4 (*p* = 0.000) accepted the hypothesis. Thus, the revised PsyCap scale had good configural and metric invariance.

**Table 5:** Results of measurement invariance assessment (*N* = 370).

Model	*χ* ^2^	*df*	*p*	CFI	TLI	RMSEA	AIC
Model 1: Configural invariance	20.037	21	0.519	0.925	0.917	0.046	1589.026
Model 2: Metric invariance	50.136	49	0.428	0.925	0.920	0.045	1563.126
Model 3: Scalar Invariance	109.657	77	0.009	0.921	0.918	0.046	1566.646
Model 4: Residual Invariance	189.879	105	0.000	0.913	0.914	0.047	1590.868

RMSEA, root-mean-square error of approximation; CFI, Comparative Fit Index; TLI, Tucker-Lewis Index; AIC, Akaike’s information criterion.

#### Validity

Convergent validity refers to the extent of the correlations between multiple indicators that measure the same latent construct (Carlson and Herdman, 2012). When all the standardized loadings of items are larger than 0.7 (*p* < 0.01), all the average variance extracted (AVE) of constructs are larger than 0.5, and all the construct reliability (CR) of constructs are larger than 0.7, the convergent validity of the scale is good (Hair et al., 2009). The results of the standardized loadings, AVE, and CR of the revised PsyCap scale are presented in Table 4. All the values of AVE were larger than 0.5, and all the values of CR were larger than 0.7. Most standardized loadings of items were larger than 0.7, while only four were nearly 0.7. Therefore, these results show that the revised PsyCap scale has good convergent validity.

Discriminant validity refers to the extent to which one construct differs from one another empirically, which is usually assessed by the Fornell-Larcker criterion (Rönkkö and Cho, 2022). When the square root of the AVE of one construct is larger than the correlations between it and other latent constructs, the discriminant validity of this construct is good (Fornell and Larcker, 1981). Therefore, this study adopted the Fornell-Larcker criterion analysis to assess the discriminant validity of seven PsyCap constructs. Results showed that all the √AVEs of optimism, resilience, wisdom, gratitude and dedication, contentment and happiness, meaning in life, and self-efficacy were larger than 0.7, and all the correlation coefficients were smaller than 0.7 (see Table 6). It means the revised PsyCap scale has good discriminant validity.

**Table 6:** Fornell-Larcker criterion analysis of the revised PsyCap scale (*N* = 370).

√AVE r	1	2	3	4	5	6	7
1. Optimism	0.832						
2. Resilience	0.520^**^	0.804					
3. Wisdom	0.361^**^	0.139^**^	0.799				
4. Gratitude and dedication	0.552^**^	0.364^**^	0.507^**^	0.723			
5. Contentment and happiness	0.595^**^	0.398^**^	0.171^**^	0.597^**^	0.798		
6. Meaning in life	0.293^**^	0.159^**^	0.444^**^	0.530^**^	0.264^**^	0.888	
7. Self-efficacy	0.630^**^	0.518^**^	0.521^**^	0.600^**^	0.384^**^	0.524^**^	0.796

*Represents a significance level of *p* < 0.05; **Represents a significance level of *p* < 0.01. √AVE, the square root of the average variance extracted; *r*, the correlations.

Criterion validity evaluates how accurately a test measures the outcome it was designed to measure. One of the simplest ways to assess criterion-related validity is to compare it to a known standard (Nikolopoulou, 2022). After controlling the demographic variables, such as age, gender, marriage, and education, the scores of PsyCap and its seven factors were positively related to social participation (*r* = 0.14 ~ 0.41, *p* < 0.01), self-rated health (*r* = 0.16 ~ 0.52, *p* < 0.01), and life satisfaction (*r* = 0.22 ~ 0.65, *p* < 0.01) of older people and negatively correlated with depression (*r* = −0.71 ~ −0.24, *p* < 0.01). In addition, the effects of total PsyCap on criterion-related variables were greater than seven factors (see Table 7). Therefore, the PsyCap scale for older people has good calibration validity.

**Table 7:** Results of correlations between PsyCaps and other variables (*N* = 370).

PsyCaps	Depression	Social participation	Self-rated health	Life satisfaction
Optimism	−0.661^**^	0.301^**^	0.485^**^	0.653^**^
Self-efficacy	−0.612^**^	0.406^**^	0.477^**^	0.527^**^
Resilience	−0.651^**^	0.279^**^	0.451^**^	0.553^**^
Wisdom	−0.238^**^	0.144^**^	0.159^*^	0.215^**^
Gratitude and dedication	−0.487^**^	0.247^**^	0.269^**^	0.531^**^
Contentment and happiness	−0.483^**^	0.243^**^	0.346^**^	0.470^**^
Meaning in life	−0.384^**^	0.290^**^	0.355^**^	0.328^**^
Total PsyCap	−0.710^**^	0.392^**^	0.521^**^	0.653^**^

*Represents a significance level of *p* < 0.05; **Represents a significance level of *p* < 0.01. Control variables: age, gender, marriage, education.

## Study 3

### Participants

This study carried out two rounds of random sampling surveys with the permission of the Civil Affairs Bureau of provincial capital cities (including Chengdu, Xi ‘an, and Kunming) in western China. All the respondents were over 60 years old and could answer all the questions. Participants provided their informed consent and volunteered to participate in this interview without compensation. This study interviewed 350 older people and screened out 328 valid questionnaires (basic information of Sample 3 see in Table 2). The average age of the subjects was 79.73 years, ranging from 60 to 99 years old. 47% of the participants were male, and 53% were male. 58.8% of the respondents are married, and 41.2% are single. As for educational level, 14.4% of participants were in undergraduate and above, 7.6% were in junior college, 14.9% were in senior high school, 13.4% were in junior high school, and 49.7% were in primary school and illiterate.

### Measurements

*Psychological capital* was measured by the revised PsyCap questionnaire constructed in Study 2, including seven dimensions. Each dimension had four self-reported questions, and every question was evaluated by a Likert 5-point scale, from ‘very disagreeable’ to ‘very agreeable.’ The Cronbach’s alpha for *psychological capital* was 0.939.

*Depression* was also measured using the shortened version of the Center for Epidemiologic Studies Depression Scale (CES-D9, Santor and Coyne, 1997). This study used a total score of nine questions to measure *depression*. The Cronbach’s alpha for *depression* was 0.858.

### Analytical approaches

The linear regression model is a common analytical method to test the linear relationship between the continuous dependent variable and the predictors (Poole and O’Farrell, 1971). Thus, this study used SPSS 21.0 software to set the linear regression model to test the relationship between depression and PsyCap.

### Results

Before conducting correlation analysis, Harman’s single-factor method was used to test whether there were common method biases between independent variables and dependent variables in Sample 3. Results showed that the percentage of variance interpretation of the first common factor was 22.62%, which was less than 40% of the critical value. Therefore, there is no serious problem of common method biases in the survey data obtained in this study.

According to the scatter point diagram results, meaning in life, contentment and happiness, optimism, resilience, wisdom, gratitude and dedication, self-efficacy, and total PsyCap had a linear relationship with depression, respectively. The results of linear regression models are presented in Table 8. After controlling the demographic variables (e.g., age, gender, marriage, and education), the total PsyCap and its seven elements were negatively correlated with depression (*r* = −0.419 ~ −0.163, *p* < 0.01). Therefore, PsyCap has a positive effect on the mental health of older people.

**Table 8:** Relationships between PsyCaps and depression (*N* = 328).

Dependent variable	Depression								
Model	1	2	3	4	5	6	7	8	9
**Control variables**									
1. Gender	−0.136^**^	−0.107^**^	−0.136^**^	−0.150^**^	−0.150^**^	−0.122^**^	−0.127^**^	−0.126^**^	−0.130^**^
2. Age	0.197^**^	0.251^*^	0.130^**^	0.226^**^	0.165^**^	0.157^**^	0.221^**^	0.137^**^	0.162^**^
3. Marriage	0.252^**^	0.166^**^	0.191^**^	0.221^**^	0.222^**^	0.209^**^	0.223^**^	0.232^**^	0.163^**^
4. Education	−0.216^**^	−0.228^**^	−0.165^**^	−0.204^**^	−0.189^**^	−0.175^**^	−0.225^**^	−0.174^**^	−0.165^**^
**Independent variables**									
1. Optimism		−0.393^**^							
2. Self-efficacy			−0.392^**^						
3. Resilience				−0.296^**^					
4. Wisdom					−0.163^**^				
5. Gratitude and dedication						−0.290^**^			
6. Contentment and happiness							−0.216^**^		
7. Meaning in life								−0.238^**^	
8. PsyCap									−0.419^**^
*R* ^2^	0.217	0.363	0.347	0.293	0.229	0.284	0.253	0.256	0.368
*F*	25.298	41.543	40.248	31.640	22.956	30.268	25.948	26.432	44.029

*Represents a significance level of *p* < 0.05; **Represents a significance level of *p* < 0.01.

## Discussion

Based on a literature review and semi-structured interviews of Chinese older people, this study developed a primary PsyCap questionnaire and subsequently collected 896 questionnaires of older people to finish EFA, CFA, and the criterion-related validity test. Results demonstrated that older people’s PsyCap is composed of meaning in life, contentment and happiness, optimism, resilience, wisdom, gratitude and dedication, and self-efficacy. The scale of the seven-factor structure included 28 items and showed good construct validity and reliability. Findings also showed that total PsyCap and its seven factors significantly improve mental health of older adults.

Compared with existing research, optimism, resilience, and self-efficacy also were found in the PsyCap among older people, which can be regarded as the cross-cultural and cross-group PsyCap. The concepts and theoretical mechanisms of optimism, resilience, and self-efficacy have been discussed much in the existing studies (Youssef-Morgan and Luthans, 2015; Luthans and Youssef-Morgan, 2017). For older people, PsyCap also has a broadening effect on positive affective states in times of adversity, cultivates positive and constructive memories that contribute to health, and facilitates positive cognitive appraisals of life events.

In addition, this study still discovered some unique elements of PsyCap among older people, including the meaning in life, contentment and happiness, gratitude and dedication, and wisdom. In other studies, these elements were also considered as internal positive psychological advantages which promote individuals’ quality of life, interpersonal relationships, and social development (Snyder and Lopez, 2001; Peterson and Seligman, 2004; Luthans et al., 2007). For older people, these unique types of PsyCap have a close connection with the characteristics of their life experiences, which can be explained by three psychological mechanisms of socioemotional selectivity theory.

First, the limitation of perceived time makes older people pay more attention to the development of self-concept than information seeking (Carstensen, 1995). The meaning in life as a critical part of self-concept plays a role in activating older people a solid motivation to survive, confirmed by some interviewed older people with diseases and disabilities. Second, the limitation of perceived time also generates positive effects on memory, which can older adults tend to remember positive information and life experiences (Charles et al., 2003). There are some similar findings in the survey that many older people deeply remember others’ help and try to transform the emotion of gratitude into positive life attitudes and behaviors. Third, the limitation of perceived time also changes the attentional content of older people, making them focus on savoring the moment instead of future goals (Carstensen and Hershfield, 2021). Most participants rarely discuss futural plans and always emphasize the happiness in their current life. It is a reasonable explanation for the replacement of ‘hope’ by ‘contentment and happiness’ in the elements of PsyCap among older people.

Finally, this study demonstrated some positive outcomes of PsyCap among older people. As the existing research suggested, PsyCap positively affects health promotion (Luthans et al., 2013; Azimi, 2014). This study also reported similar findings that PsyCap improves the satisfaction of physical health and decreases the depression of older people. On the other hand, the positive effects of PsyCap on individual development have been proved, such as promoting interpersonal relationships, working performance, and volunteering behaviors (Newman et al., 2014; Krasikova et al., 2015; Xu et al., 2021). In this study, this positive effect is manifested by promoting the social participation of older people, including formal and informal social activities. At last, this study still found that PsyCap has a significant impact on increasing the life satisfaction of older people, which is similar to other studies (Avey et al., 2010; Jafari and Hesampour, 2017). In conclusion, PsyCap positively impacts many aspects of older people.

Some shortcomings of this study need to be noted. First, thanks to the epidemic of COVID-19 and the lack of older adults’ contact information, this study cannot conduct a second follow-up survey to examine the PsyCap scale’s test–retest reliability and measurement invariance across different times. Second, this study focused on Chinese older people, so the applicability of the PsyCap scale needs to be tested across different ethnicities and cultures. Third, the lack of a longitudinal data limited the study on causality between PsyCap and mental health. Fourth, this study adopted self-reported data, which may cause the results to be in bias. These limitations indicate towards future research possibilities.

## Conclusion

Notwithstanding the previously mentioned limitations, this study adds to the literature on older adults’ health promotion and social development from the perspective of positive advantages. As advocated by the Health Ageing Strategy of World Health Organization (2015), maintaining and promoting the intrinsic ability of older people is the basis of functional development. As a part of the inherent ability, the PsyCap of older people contains a variety of positive psychological resources and advantages, which can activate their health capital, human capital, and social capital to realize full development. This study developed a seven-factor PsyCap scale covering 28 items, which provides a reliable quantitative research tool for subsequent psychosocial science research of older people. Exploring cross-cultural implications, antecedents, outcomes, and interventions of the PsyCap among older people should be the focus of future research.

## Data availability statement

The raw data supporting the conclusions of this article will be made available by the authors, without undue reservation.

## Ethics statement

The studies involving human participants were reviewed and approved by Humanities Research Ethics Review Committee of Sichuan University. The patients/participants provided their written informed consent to participate in this study.

## Author contributions

YX: data collection, data analysis, and paper writing. DL: research design and paper revision. All authors contributed to the article and approved the submitted version.

## Funding

This work was supported by Sichuan University under Grant number 19HHF-05.

## Conflict of interest

The authors declare that the research was conducted in the absence of any commercial or financial relationships that could be construed as a potential conflict of interest.

## Publisher’s note

All claims expressed in this article are solely those of the authors and do not necessarily represent those of their affiliated organizations, or those of the publisher, the editors and the reviewers. Any product that may be evaluated in this article, or claim that may be made by its manufacturer, is not guaranteed or endorsed by the publisher.

## References

[ref2] Ashraf F. Khan M. A. (2017). Broadening the positive psychological capital construct: an Asian cultural perspective. J. Independent Stud. Res. Manage. Soc. Sci. Econ. 15, 91–105. doi: 10.31384/jisrmsse/2017.15.2.7

[ref3] Avey J. B. Luthans F. Smith R. M. Palmer N. F. (2010). Impact of positive psychological capital on employee well-being over time. J. Occup. Health Psychol. 15, 17–28. doi: 10.1037/a0016998, PMID: 20063956

[ref4] Avey J. B. Reichard R. J. Luthans F. Mhatre K. H. (2011). Meta-analysis of the impact of positive psychological capital on employee attitudes, behaviors, and performance. Hum. Resour. Dev. Q. 22, 127–152. doi: 10.1002/hrdq.20070

[ref5] Azimi T. (2014). The role of psychological capitals in predicting mental health and well-being of female employees in the education of dehdasht. Indian J. Fundam. Appl. Life Sci. S4, 1297–1304.

[ref6] Bedaso T. S. Han B. (2021). Attitude toward aging mediates the relationship between personality and mental health in older adults. Healthcare 9:594. doi: 10.3390/healthcare9050594, PMID: 34067910 PMC8156287

[ref7] Bin W. Zhu L. (2020). The research on the connotation and structure of Chinese college Teachers' psychological capital. Int. J. Inf. Educ. Technol. 10, 460–465. doi: 10.18178/ijiet.2020.10.6.1407

[ref8] Bockorny K. Youssef-Morgan C. M. (2019). Entrepreneurs’ courage, psychological capital, and life satisfaction. Front. Psychol. 10:789. doi: 10.3389/fpsyg.2019.00789, PMID: 31024410 PMC6461011

[ref9] Brown T. A. Moore M. T. (2012). “Confirmatory factor analysis,” in Handbook of structural equation modeling. ed. Hoyle R. H. (New York: Guilford press), 361–379.

[ref10] Carlson K. D. Herdman A. O. (2012). Understanding the impact of convergent validity on research results. Organ. Res. Methods 15, 17–32. doi: 10.1177/1094428110392383

[ref11] Carstensen L. L. (1995). Evidence for a life-span theory of socioemotional selectivity. Curr. Dir. Psychol. Sci. 4, 151–156. doi: 10.1111/1467-8721.ep11512261 PMC8340497 34366582

[ref12] Carstensen L. L. Hershfield H. E. (2021). Beyond stereotypes: using socioemotional selectivity theory to improve messaging to older adults. Curr. Dir. Psychol. Sci. 30, 327–334. doi: 10.1177/09637214211011468, PMID: 34366582 PMC8340497

[ref13] Charles S. T. Mather M. Carstensen L. L. (2003). Aging and emotional memory: the forgettable nature of negative images for older adults. J. Exp. Psychol. Gen. 132, 310–324. doi: 10.1037/0096-3445.132.2.310, PMID: 12825643

[ref14] Cheung F. Lucas R. E. (2014). Assessing the validity of single-item life satisfaction measures: results from three large samples. Qual. Life Res. 23, 2809–2818. doi: 10.1007/s11136-014-0726-4, PMID: 24890827 PMC4221492

[ref16] Cole K. (2006). Wellbeing, Psychological Capital, and Unemployment: An Integrated Theory. In Joint Annual Conference of the International Association for Research in Economic Psychology and the Society for the Advancement of Behavioral Economics. (pp. 5–8). Paris, France.

[ref17] Corbin J. M. Strauss A. (1990). Grounded theory research: procedures, canons, and evaluative criteria. Qual. Sociol. 13, 3–21. doi: 10.1007/BF00988593

[ref18] Cordaro D. T. Brackett M. Glass L. Anderson C. L. (2016). Contentment: perceived completeness across cultures and traditions. Rev. Gen. Psychol. 20, 221–235. doi: 10.1037/gpr0000082

[ref19] Dhir R. Sharma V. (2020). Exploring dimensions of psychological capital through grounded theory investigations. Int. J. Indian Cult. Bus. Manage. 20, 109–132. doi: 10.1504/IJICBM.2020.105556

[ref20] Donaldson S. I. Chan L. B. Villalobos J. Chen C. L. (2020). The generalizability of hero across 15 nations: positive psychological capital (psycap) beyond the us and other weird countries. Int. J. Environ. Res. Public Health 17:9432. doi: 10.3390/ijerph17249432, PMID: 33339210 PMC7765579

[ref21] Emmons R. A. Froh J. Rose R. (2019). “Gratitude” in Positive Psychological Assessment: A Handbook of Models and Measures. eds. Gallagher M. W. Lopez S. J. (Washington, DC: American Psychological Association), 317–332.

[ref22] Erikson E. H. Erikson J. M. Kivnick H. Q. (1994). Vital Involvement in Old Age. New York: WW Norton & Company.

[ref23] Fabrigar L. R. Wegener D. T. (2011). Exploratory Factor Analysis. Oxford: Oxford University Press.

[ref24] Fornell C. Larcker D. F. (1981). Evaluating structural equation models with unobservable variables and measurement error. J. Mark. Res. 18, 39–50. doi: 10.1177/002224378101800104

[ref26] Goldsmith A. H. Veum J. R. Darity W. Jr. (1997). The impact of psychological and human capital on wages. Econ. Inq. 35, 815–829. doi: 10.1111/j.1465-7295.1997.tb01966.x

[ref27] Hagestad G. O. Uhlenberg P. (2005). The social separation of old and young: a root of ageism. J. Soc. Issues 61, 343–360. doi: 10.1111/j.1540-4560.2005.00409.x

[ref28] Hair J. F. Black W. C. Babin B. J. Anderson R.E. (2009). Multivariate Data Analysis (7th). Upper Saddle River: Prentice Hall

[ref29] Howrey B. T. Hand C. L. (2019). Measuring social participation in the health and retirement study. Gerontologist 59, e415–e423. doi: 10.1093/geront/gny094 30169644

[ref30] Jafari A. Hesampour F. (2017). Predicting life satisfaction based on spiritual intelligence and psychological capital in older people. Iranian J. Ageing 12, 90–103. doi: 10.21859/sija-120190

[ref31] Ke J. L. Sun J. M. Li Y. R. (2009). Psychological capital: Chinese indigenous scale’s development and its validity comparison with the western scale. Acta Psychol. Sin. 41, 875–888. doi: 10.3724/SP.J.1041.2009.00875

[ref32] Kim M. Oja B. D. Anagnostopoulos C. (2021). An expanded psychological capital (A-HERO) construct for creativity: building a competitive advantage for sport organizations. Eur. Sport Manag. Q., 1–23. doi: 10.1080/16184742.2021.1922480

[ref33] Krasikova D. V. Lester P. B. Harms P. D. (2015). Effects of psychological capital on mental health and substance abuse. J. Leaders. Organ. Stud. 22, 280–291. doi: 10.1177/1548051815585853

[ref34] Letcher L. (2003). Psychological Capital and Wages: A Behavioral Economic Approach. Kansas: Kansas State University.

[ref35] Luthans F. (2002). The need for and meaning of positive organizational behavior. J. Organ. Behav. 23, 695–706. doi: 10.1002/job.165

[ref36] Luthans F. Avolio B. J. Walumbwa F. O. Li W. (2005). The psychological capital of Chinese workers: exploring the relationship with performance. Manag. Organ. Rev. 1, 249–271. doi: 10.1111/j.1740-8784.2005.00011.x

[ref37] Luthans F. Youssef C. M. Avolio B. J. (2007). Psychological Capital: Developing the Human Competitive Edge. Oxford: Oxford university press.

[ref38] Luthans F. Youssef C. M. Sweetman D. S. Harms P. D. (2013). Meeting the leadership challenge of employee well-being through relationship PsyCap and health PsyCap. J. Leadersh. Organ. Stud. 20, 118–133. doi: 10.1177/1548051812465893

[ref39] Luthans F. Youssef-Morgan C. M. (2017). Psychological capital: an evidence-based positive approach. Annu. Rev. Organ. Psych. Organ. Behav. 4:339. doi: 10.1146/annurev-orgpsych-032516-113324

[ref40] Meng Q. Xie Z. Zhang T. (2014). A single-item self-rated health measure correlates with objective health status in the elderly: a survey in suburban Beijing. Front. Public Health 2:27. doi: 10.3389/fpubh.2014.00027, PMID: 24783187 PMC3989711

[ref41] Murniati N. Al Aufa B. Kusuma D. Kamso S. (2022). A scoping review on biopsychosocial predictors of mental health among older adults. Int. J. Environ. Res. Public Health 19:10909. doi: 10.3390/ijerph191710909, PMID: 36078627 PMC9518331

[ref42] Newman A. Ucbasaran D. Zhu F. E. I. Hirst G. (2014). Psychological capital: a review and synthesis. J. Organ. Behav. 35, S120–S138. doi: 10.1002/job.1916

[ref43] Nikolopoulou K. (2022). What Is Criterion Validity?|Definition & Examples. Available at: https://www.scribbr.com/methodology/criterion-validity/ (Accessed October 21, 2022).

[ref44] Nolzen N. (2018). The concept of psychological capital: a comprehensive review. Manage. Rev. Q. 68, 237–277. doi: 10.1007/s11301-018-0138-6

[ref45] Peterson C. Seligman M. E. (2004). Character Strengths and Virtues: A Handbook and Classification. Oxford: Oxford University Press.

[ref46] Polit D. F. Beck C. T. (2010). Essentials of Nursing Research: Appraising Evidence for Nursing Practice. Philadelphia: Lippincott Williams & Wilkins.

[ref47] Poole M. A. O’Farrell P. N. (1971). The assumptions of the linear regression model. Trans. Inst. Br. Geogr. 145-158:145. doi: 10.2307/621706

[ref48] Rahimnia F. Mazidi A. Mohammadzadeh Z. (2013). Emotional mediators of psychological capital on well-being: the role of stress, anxiety, and depression. Manage. Sci. Lett. 3, 913–926. doi: 10.5267/j.msl.2013.01.029

[ref49] Ren Y. Ji B. (2019). Correlation between perceived social support and loneliness among Chinese adolescents: mediating effects of psychological capital. Psychiatr. Danub. 31, 421–428. doi: 10.24869/psyd.2019.421, PMID: 31698398

[ref50] Riley M. W. Riley J. W. (1994). Age integration and the lives of older people. The Gerontologist 34, 110–115. doi: 10.1093/geront/34.1.110 8150298

[ref52] Roche M. Haar J. M. Luthans F. (2014). The role of mindfulness and psychological capital on the well-being of leaders. J. Occup. Health Psychol. 19, 476–489. doi: 10.1037/a0037183, PMID: 24933594

[ref53] Rönkkö M. Cho E. (2022). An updated guideline for assessing discriminant validity. Organ. Res. Methods 25, 6–14. doi: 10.1177/10944281209686

[ref54] Santor D. A. Coyne J. C. (1997). Shortening the CES–D to improve its ability to detect cases of depression. Psychol. Assess. 9, 233–243. doi: 10.1037/1040-3590.9.3.233

[ref55] Sargent-Cox K. A. Anstey K. J. Luszcz M. A. (2012). Change in health and self-perceptions of aging over 16 years: the role of psychological resources. Health Psychol. 31, 423–432. doi: 10.1037/a0027464, PMID: 22429127

[ref56] Schmitt N. Kuljanin G. (2008). Measurement invariance: review of practice and implications. Hum. Resour. Manag. Rev. 18, 210–222. doi: 10.1016/j.hrmr.2008.03.003

[ref58] Shah S. K. Corley K. G. (2006). Building better theory by bridging the quantitative–qualitative divide. J. Manag. Stud. 43, 1821–1835. doi: 10.1111/j.1467-6486.2006.00662.x

[ref59] Snyder C. R. Lopez S. J. (2001). Handbook of Positive Psychology. Oxford: Oxford University Press.

[ref60] Stajkovic A. D. Luthans F. (1998). Social cognitive theory and self-efficacy: going beyond traditional motivational and behavioral approaches. Organ. Dyn. 26, 62–74. doi: 10.1016/S0090-2616(98)90006-7

[ref61] Steger M. F. Frazier P. Oishi S. Kaler M. (2006). The meaning in life questionnaire: assessing the presence of and search for meaning in life. J. Couns. Psychol. 53, 80–93. doi: 10.1037/0022-0167.53.1.80

[ref62] Steptoe A. O'Donnell K. Marmot M. Wardle J. (2008). Positive affect, psychological well-being, and good sleep. J. Psychosom. Res. 64, 409–415. doi: 10.1016/j.jpsychores.2007.11.008, PMID: 18374740

[ref63] Tucker L. R. MacCallum R. C. (1997). Exploratory Factor Analysis. Columbus: Ohio State University, Columbus.

[ref64] Webster J. D. (2003). An exploratory analysis of a self-assessed wisdom scale. J. Adult Dev. 10, 13–22. doi: 10.1023/A:1020782619051

[ref65] Wells Y. D. Kendig H. L. (1999). Psychological resources and successful retirement. Aust. Psychol. 34, 111–115. doi: 10.1080/00050069908257438

[ref66] Windle G. Markland D. A. Woods R. T. (2008). Examination of a theoretical model of psychological resilience in older age. Aging Ment. Health 12, 285–292. doi: 10.1080/13607860802120763, PMID: 18728940

[ref67] Windsor T. D. Curtis R. G. Luszcz M. A. (2015). Sense of purpose as a psychological resource for aging well. Dev. Psychol. 51, 975–986. doi: 10.1037/dev0000023, PMID: 26010384

[ref68] World Health Organization (2015). World report on ageing and health. Geneva: World Health Organization.

[ref69] Xu L. P. Liao J. B. Wu Y. S. Kuang H. D. (2021). Effect of psychological capital of volunteers on volunteering behavior: the chained mediation role of perceived social support and volunteer motivation. Front. Psychol. 12:657877. doi: 10.3389/fpsyg.2021.657877, PMID: 34603118 PMC8484802

[ref70] Xu C. Yu H. (2019). “The relationship between disabled college students’ psychological capital and employability” in 2019 International Conference on Advanced Education and Social Science Research (ICAESSR 2019). eds. Balakrishnan S. Chung M. A. (Paris: Atlantis Press), 129–132.

[ref72] Youssef-Morgan C. M. Luthans F. (2015). Psychological capital and well-being. Stress Health 31, 180–188. doi: 10.1002/smi.2623 26250352

[ref73] Zhang K. Zhang S. Dong Y. (2010). Positive psychological capital: measurement and relationship with mental health. Stud. Psychol. Behav. 1, 58–64.

